# Association between perceived neighborhood environment and health of middle-aged women living in rapidly changing urban Mongolia

**DOI:** 10.1186/s12199-017-0659-y

**Published:** 2017-05-31

**Authors:** Tserendulam Shagdarsuren, Keiko Nakamura, Layla McCay

**Affiliations:** 1 Department of Global Health Entrepreneurship, Division of Public Health, Graduate School of Medical and Dental Sciences, Tokyo Medical and Dental University, Yushima 1-5-45, Bunkyo-ku, Tokyo, 113-8519 Japan; 2 Promotion Committee for Healthy Cities, Tokyo, Japan; 3 Centre for Urban Design and Mental Health, London, UK

**Keywords:** Urbanization, Neighborhood environment, General health, Mental health, Middle-aged women, Mongolia

## Abstract

**Background:**

This study was conducted in rapidly urbanizing Ulaanbaatar, Mongolia, to examine patterns of perceived neighborhood quality by residents and the associations between these patterns and self-reported general and mental health in middle-aged women.

**Methods:**

A questionnaire survey was administered to 960 women aged 40–60 years. Demographic and socio-economic characteristics, subjects’ perception of their neighborhood environment, general health status, and mental health as measured using a 12-item General Health Questionnaire (GHQ12) were reported.

**Results:**

A total of 830 women completed the questionnaire. Subjects reporting their general health as very good or good accounted for 80.3% and those with a GHQ12 ≥16, which reflects psychological distress or severe distress, accounted for 16.1%. A principal component analysis of the perceptions of neighborhood environment by the residents identified six qualities: physical environment, designed environment, neighborhood community, public safety, natural environment, and citizen services. The perception of better-quality citizen services in the neighborhood was associated with better self-reported general health (odds ratio [OR] = 1.330, 95% confidence interval [CI] 1.093–1.618), and the perception of better-quality public safety was associated with less psychological distress (OR = 0.718, 95% CI 0.589–0.876); these associations were independent of education, income, occupation, type of residential area, and number of years living in the current *khoroo*.

**Conclusions:**

The perception of the quality of a neighborhood environment can affect the self-reported general and mental health of residents, even after accounting for the type of residential area and individual socio-economic status. Developing high-quality neighborhoods is an essential component of good planning to promote population health in urban environments.

## Background

Ulaanbaatar, the capital city of Mongolia, has experienced rapid economic and population growth during the last 15 years [1]. Between 2000 and 2015, the city’s gross domestic product (GDP) grew by 24 times to 6.6 million US dollars (USD); during that same period, the population grew by 80%. Ulaanbaatar is now home to 1.4 million people. The city presently faces several economic challenges: because of the country’s dependence on its mining industry, fluctuations in world prices for commodities are destabilizing the economy; nevertheless, the government maintains increasingly expansionary fiscal policies, and a high inflation rate (averaging 11.2% from 2007 to 2015) has been reported [1]. Because of this level of population and economic growth and the accompanying challenges, urban planning is now a priority to ensure the delivery of affordable, quality housing and favorable living environments.

The *ger* residence, which is a combination of traditional nomadic tents and small houses with limited access to urban infrastructure, and Russian-style apartment residences, which were established between 1960 and 1980, were the two major types of residences in Ulaanbaatar in the early 2000s. Since the late 2000s, however, the redevelopment of existing housing areas has led to the emergence of new town areas consisting of comfortable, detached houses with modern amenities to accommodate the growing number of middle- and upper-class families. These new towns have been established only 5–10 km from the city center, are distant from city noise, and provide a quiet, natural-feeling atmosphere with less air pollution in winter, compared with other residential areas [2]. In the past, summer residences located in the city’s outskirts were only used during the summer time. Because of the recent progress in infrastructure development, however, some people now choose to live in summer house areas throughout the year. Although only 0.3% of the households in Ulaanbaatar were living in houses in the new town areas in 2010, the Master Plan Agency of Ulaanbaatar forecasts that this percentage will increase to 16.7% by the year 2030 [3].

With the urban residential environment in Ulaanbaatar undergoing these rapid changes, many citizens have sought to upgrade their housing and neighborhood; as they do so, their neighborhood environment, including both physical and social environmental aspects, has been evolving. The impact of these changes on the health of urban dwellers is a concern. Studies have discussed the association between the physical and social environments of neighborhoods and the health of their residents. In particular, residents’ perceptions of their neighborhood environment might be associated with their health [4, 5]. While some evidence of a correlation between social capital and health in low-middle income countries has been accumulated [6], other aspects of the correlation between neighborhood environment and health have not been well discussed in low–middle-income countries.

The life expectancy of the Mongolian population has been extended over the last 20 years, and the life expectancy of women at birth (75.8 years) is longer than the life expectancy of men (66.0 years) [1]. More women than men are living into middle age and old age. Women in Mongolia tend to attain a higher level of education than men [7] and to assert both leadership and caregiving roles in community life [8]. As such, programs for middle-aged women that promote health and well-being may leverage leadership and caregiving roles that will benefit the health of their families and community [9–11]. These women’s perceptions of their environment and health in Ulaanbaatar will help to target urban health development effectively in this rapidly growing city.

The city of Ulaanbaatar is an interesting setting in which to consider how living in a rapidly developing and urbanizing community affects its population’s health, within the context of a city and country’s social and economic development. As these transitions reshape communities’ neighborhood physical and social environments, the question naturally emerges: how do neighborhood qualities affect people’s general and mental health in the city?

The objectives of this study were (1) to examine patterns of perceived neighborhood quality by residents and (2) to elucidate relationships between these perceived neighborhood qualities and the self-reported general and mental health of middle-aged women living in Ulaanbaatar.

## Methods

### Study area and subjects

The study was conducted in Ulaanbaatar, the capital city of Mongolia. Subjects were selected from four types of residential areas: summer house areas, *ger* areas, apartment areas, and luxury residential areas in Ulaanbaatar, Mongolia. The summer house area is a recently developed Mongolian traditional residence area characterized by the exposure of the residences to a natural setting in the city’s peri-urban green zone. The *ger* area describes the Mongolian traditional residential area in the central and middle areas of the city, which have access to electricity but lack a drinking water supply, a hot water supply, heating, toilets, and a bath/shower room. Buildings in the *ger* area are mixture of *ger* tents and small houses. The apartment area was established between 1960 and 1980 and consists of apartment buildings, mostly Russian-style apartments, that have access to central heating, a water supply, and a sanitation system; these apartments are also located in the central and middle areas of the city. The luxury residential area is a quiet, newly developed area near the city center with comfortable houses and luxury apartment buildings; it has been developed since 2010.

There were 152 *khoroos* (administrative units) in Ulaanbaatar, divided into 3 *khoroos* for summer house areas, 72 *khoroos* for *ger* areas, 49 *khoroos* for apartment areas, and 2 *khoroos* for luxury residential areas, according to the major area type within each *khoroos*. The rest of 29 *khoroos* were characterized with mixture of area types and they were excluded from the study *khoroos*. A total of 12 *khoroos* (2 *khoroos* for summer house areas, 4 *khoroos* for *ger* areas, 4 *khoroos* for apartment areas, and 2 *khoroos* for luxury residential areas) were selected by stratified random sampling and assigned as study *khoroos*. First stratification was according to area types (summer house, *ger*, apartment, and luxury residential), and the second stratification was according to the estimated population of women aged 40–60 (population of middle-aged women ≥1500; 1000–1500; 500–1000; <500). Randomly selected 2 *khoroos* from 3 *khoroos* for summer house areas and both 2 *khoroos* of luxury residential areas were assigned as study *khoroos*. This disproportionate sampling of *khoroos* was conducted by assuming that the proportion of summer houses and luxury residential areas will continue to grow over the next few years, and in-depth observations of these newly emerging areas was thought to be necessary in this study.

The sample size required for the population 162,300, with a confidence interval of 3.5 and confidence level of 95% was determined to be 780. By dividing this sample size into 12 *khoroos*, to have minimum 65 subjects in each *khoroo*, 80 female residents were randomly selected from each of the 12 *khoroos*; thus, a total of 960 samples (160 for summer house areas, 320 for *ger* areas, 320 for apartment areas, and 160 for luxury residence areas) were examined. Using the registration records of the *khoroo* offices, one in every ten households was randomly selected, and then one of the women registered as living in that household and who was between the ages of 40 and 60 years old was identified as potential subjects. This selection process was repeated until 80 female residents were listed for one *khoroo*. Only one eligible woman aged 40–60 years old was included from each household. After inclusion on the list of potential subjects, health records at the *khoroo* health center, report from the family, or reports from subjects themselves on their physical condition were then used to exclude women with severe illness or physical and/or mental disabilities from the interview and examination.

### Survey procedure

Between August and September 2015, six teams were assigned to conduct interviews with the subjects in their houses. A team of three women—one female data collector (a surveyor from the National Public Health Institute of Mongolia), one female data collection assistant, and one female appointed officer of the relevant *khoroo* office—met each woman on the list and conducted face-to-face interviews using structured questionnaire forms. The *khoroo* offices helped the teams to make the initial contact with each woman. Four male assistants provided help with logistics and security during the survey. Written informed consent was obtained before the start of the interview. The interview team members participated in a 1-day training workshop in advance to develop an understanding of the objectives and the study protocol and to obtain the appropriate knowledge and skills required to conduct the interview survey. The questionnaire form in the Mongolian language was pre-tested in the field to ensure that all the questions could be understood clearly.

### Study variables

#### Demographic and socio-economic characteristics

Age, marital status, education level, monthly household income, housing types, occupation, and living years in the current *khoroo* were recorded. These variables were included in the analysis because they were assumed to be associated with the general and mental health of residents and to exhibit potential variations across residence areas. Education level was classified into five categories according to the highest level of school that the subjects had completed (basic education, high school education, vocational education, college or university level education, and graduate school education), and the household income was divided into five categories (<500,000 Mongolian Tugrik (MNT) [<250 USD]; ≥500,000 and <800,000 MNT [≥250 and <400 USD]; ≥800,000 and <1,200,000 MNT [≥400 and <600 USD]; ≥1,200,000 and <2,500,000 MNT [≥600 and <1240 USD]; and ≥2,500,000 MNT [≥1240 USD]) according to reports of monthly income. Housing types were categorized into four categories: *ger* tent, small house in a *ger* area, apartment flat, and comfortable detached house. A *ger* tent is made from a wooden frame and covered by wool felt and includes all types of Mongolian traditional nomadic and deer-breeder’s tents. A small house in a *ger* area is a small house with one or more rooms with an individually fenced yard in a *ger* area. The facilities are limited and the small house does not have a bathroom, showers, central heating, sanitation, or toilet. An apartment flat is a unit in an apartment building and consists of one or more rooms with a toilet, shower or bathroom, and kitchen. It has access to electricity, heating, water, and sanitation systems. A comfortable detached house is defined as a 1- or 2-story house comprised of living rooms, bedrooms, kitchen, toilet, bathroom, and storage room with or without a mansard roof. It is equipped with self-sustaining heating and has access to electricity, water, sanitation system, landline telephone, and cable network services. Occupation was classified into the six categories (agriculture, forestry, fishing, gathering, hunting; mining, quarrying, refining; labor and factory work; service work; unemployed; and pensioner). Living years in the current *khoroo* was divided into three categories (less than 1 year, 1–5 years, and 6 years or longer).

#### Perception of neighborhood environment

To describe their perceptions of their neighborhood environments, the subjects were asked to indicate the extent to which they agreed with the following 23 statements: air is clean during the summer season; streets are clean; easy to find green open spaces; streets are easy to walk on; soil is safe; air is clean during the winter season; my neighborhood is friendly for children; my neighborhood is friendly for older and disabled people; there is enough car parking near my home; it is safe to come home after dark; neighbors are friendly; neighborhood schools provide standard education; my neighborhood is peaceful; my neighbors generally have good jobs; traffic is not bad; drinking water is safe; safe, healthy food is easily accessible; crime rates are low; people follow public rules; river water is clean; nature is well preserved; family health center is very close to home; and *khoroo* office provides opportunities for participation plans/events. The list of questions was developed based on questionnaires used in earlier studies [12, 13] by considering the meaning of neighborhood environments in Ulaanbaatar. The respondents were asked to score each item on a scale of 1–4 (strongly disagree, disagree, agree, and strongly agree).

#### Health variables

The self-reported overall rating of the current general health status was assessed by asking the subjects to answer the question “How do you judge your current health status?” by choosing one of four response categories: very good, good, fair, and poor. This reflects the health conditions over several months, rather than acute medical problems. The mental health of the subjects was assessed using the General Health Questionnaire 12-item version (GHQ12). The GHQ12 is an internationally validated self-reported questionnaire with 12 questions examining current psychological well-being using a 0–3 Likert scale [14]. The sum of all the items was used as a continuous score and was named the GHQ12 score, with a higher score reflecting poorer mental health. A binary scale was also applied by dividing the respondents into two groups, with a GHQ12 score ≥16 regarded as psychological distress or severe distress [15].

### Statistical analysis

The demographic and socio-economic characteristics as well as the self-reported general health and mental health, as measured using the GHQ12 of the subjects were calculated according to the four types of residential areas: summer house area, *ger* area, apartment area, and luxury residential area. The differences according to residential area were tested using the chi-square test.

A principal component analysis was performed to extract factors that independently represent particular components of the residents’ perceptions based on the subjects’ responses to the 23 questions on different aspects of their neighborhood environments. A confirmatory factor analysis was also performed to examine the goodness of fit of the model. Interpretations of the meaning of individual factors were made by two of the authors, one who was familiar with the environment in Mongolia and the other who was knowledgeable about statistics, and individual factors were labeled with representing names based on the consensus of the two authors. Factor scores for individual components were used as specific variables of individual perception of neighborhood environment quality.

Variations in the factor scores of individual components of perceptions of the neighborhood environment quality were analyzed according to the residential area, education, and household income categories by calculating the mean and SD and performing a one-way ANOVA analysis.

Associations between perceived neighborhood environment and self-reported general health (binary scale) were identified using logistic regression analyses. Associations between perceived neighborhood environment and self-reported mental health were identified using a generalized linear regression analysis (Likert scale score of GHQ12) and a logistic regression analysis (binary scale, GHQ12 score ≥16). We entered each factor into the unadjusted and adjusted models separately.

The statistical analysis was performed using SPSS, version 20, and SPSS AMOS, version 24, for Windows (IBM Corp., Armonk, NY, USA).

## Results

In total, 830 women completed the questionnaire, yielding a response rate of 86.5%. The following reasons were determined for the 130 subjects who did not complete the survey: severe illness and/or disabilities (*n* = 5), inability to devote sufficient time to the interview and examination (*n* = 16), cultural taboos against physical contact by researchers with the head and shoulders of the subjects during the taking of anthropometric measurements (*n* = 20), concerns about leakage of personal information gathered during the interview and anthropometric measurements (*n* = 29), unspecified reasons (*n* = 28), the subject could not be located at the stated residential address (*n* = 14), and other reasons (*n* = 18).

Table 1 shows the demographic and socio-economic characteristics as well as the self-reported general health and mental health of the subjects according to residential area. The mean age of the respondents was 49.2 years (SD, 6.3 years). One third of the respondents had received a college or university level of education, and women living in apartment areas (67.9%) and luxury residential areas (92.6%), in particular, had received education beyond high school. Regarding household income, 48.7% of the study population had a household monthly income of 250–600 USD. Among the subjects who lived in the luxury residential area, 92.0% of them reported a monthly household income of 600 USD or more, which was double the income of households living in summer house areas or *ger* areas. The house types in which the respondents lived reflected their residential area. The majority of house types in the summer house area and *ger* area were *ger* or small houses. The predominant house type in the apartment area was an apartment flat, while both apartment flats and comfortable detached houses were found in the luxury residential areas. The occupation of 46.0% of the subjects was self-employed service workers or service sector employees, followed by unemployment 27.7% and labor and factory work (16.2%). Pensioners comprised 7.1% of the respondents. The distribution of occupation varied according to the residential area. The percentage of service workers was highest in the luxury residential area and lowest in the summer house area. Regarding the number of years spent living in the current *khoroo*, the percentages of respondents living in their current *khoroo* for less than 1 year ranged from 1.8 to 5.6%.

**Table 1:** Demographic and socio-economic characteristics and health status of the subjects according to residential area

	All (*N* = 830)	Residential area				*P* ^a^
		Summer house area (*N* = 135)	*Ger* area (*N* = 276)	Apartment area (*N* = 269)	Luxury residential area (*N* = 150)	
	*n* (%)	*n* (%)	*n* (%)	*n* (%)	*n* (%)	
Age group						0.835
40–45 years old	288 (34.9)	41 (30.8)	105 (38.3)	88 (32.7)	54 (36.2)	
46–50 years old	188 (22.8)	28 (21.1)	61 (22.3)	65 (24.2)	34 (22.8)	
51–55 years old	188 (22.8)	34 (25.6)	60 (21.9)	59 (21.9)	35 (23.5)	
56–60 years old	161 (19.5)	30 (22.6)	48 (17.5)	57 (21.2)	26 (17.4)	
Missing	5	2	2	0	1	
Marital status						0.001
Never been married	12 (1.4)	1 (0.7)	2 (0.7)	4 (1.5)	5 (3.4)	
Married	645 (77.8)	106 (78.5)	197 (71.4)	227 (84.4)	115 (77.2)	
Separated or divorced	57 (6.9)	4 (3.0)	21 (7.6)	16 (5.9)	16 (10.7)	
Widowed	98 (11.8)	18 (13.3)	45 (16.3)	22 (8.2)	13 (8.7)	
Cohabitating	17 (2.1)	6 (4.4)	11 (4.0)	0 (0)	0 (0)	
Missing	1	0	0	0	1	
Education						<0.001
Basic education	114 (13.8)	28 (20.9)	56 (20.3)	29 (10.8)	1 (0.7)	
High school education	207 (25.0)	51 (38.1)	89 (32.2)	57 (21.3)	10 (6.7)	
Vocational education	131 (15.8)	23 (17.2)	60 (21.7)	41 (15.3)	7 (4.7)	
College or university level education	285 (34.5)	29 (21.6)	64 (23.2)	115 (42.9)	77 (51.7)	
Graduate school	90 (10.9)	3 (2.2)	7 (2.5)	26 (9.7)	54 (36.2)	
Missing	3	1	0	1	1	
Household income, month						<0.001
<500,000 MNT (<250 USD)	176 (21.3)	48 (35.6)	91 (33.1)	36 (13.4)	1 (0.7)	
≥500,000 and <800,000 MNT (≥250 and <400 USD)	224 (27.1)	45 (33.3)	111 (40.4)	61 (22.8)	7 (4.7)	
≥800,000 and <1,200,000 MNT (≥400 and <600 USD)	179 (21.6)	34 (25.9)	56 (20.4)	84 (31.3)	4 (2.7)	
≥1,200,000 and <2,500,000 MNT (≥600 and <1240 USD)	165 (19.9)	6 (4.4)	16 (5.8)	82 (30.6)	61 (40.7)	
≥2,500,000 MNT (≥1240 USD)	84 (10.1)	1 (0.7)	1 (0.4)	5 (1.9)	77 (51.3)	
Missing	2	0	1	1	0	
Housing type						<0.001
*Ger* tent	117 (14.1)	46 (34.1)	71 (25.7)	0 (0)	0 (0)	
Small house in *ger* area	283 (34.1)	85 (63.0)	198 (71.7)	0 (0)	0 (0)	
Apartment flat	397 (47.8)	1 (0.7)	7 (2.5)	268 (99.6)	121 (80.7)	
Comfortable detached house	33 (4.0)	3 (2.2)	0 (0)	1 (0.4)	29 (19.3)	
Occupation						<0.001
Agriculture, forestry, fishing, gathering, hunting	9 (1.1)	5 (3.7)	3 (1.1)	1 (0.4)	0 (0.0)	
Mining, quarrying, refining	16 (1.9)	3 (2.2)	8 (2.9)	4 (1.5)	1 (0.7)	
Labor and factory workers	133 (16.2)	22 (16.3)	76 (27.7)	30 (11.2)	5 (3.5)	
Self-employed service workers or service sector employees	378 (46.0)	41 (30.4)	91 (33.2)	145 (53.9)	101 (70.1)	
Unemployed	228 (27.7)	53 (39.3)	85 (31.0)	66 (24.5)	24 (16.7)	
Pensioner	58 (7.1)	11 (8.1)	11 (4.0)	23 (8.6)	13 (9.0)	
Missing	8	0	2	0	6	
Number of years living in the current *khoroo*						<0.001
Less than one year	27 (3.3)	3 (2.2)	5 (1.8)	15 (5.6)	4 (2.8)	
1–5 years	231 (28.1)	24 (17.8)	35 (12.8)	82 (30.5)	90 (62.5)	
6 years or longer	564 (68.6)	108 (80.0)	234 (85.4)	172 (63.9)	50 (34.7)	
Self-reported general health						<0.001
Very good	48 (5.8)	10 (7.4)	10 (3.6)	11 (4.1)	17 (11.4)	
Good	616 (74.5)	93 (68.9)	206 (74.9)	197 (73.5)	120 (80.5)	
Fair	157 (19.0)	31 (23.0)	56 (20.4)	59 (22.0)	11 (7.4)	
Poor	6 (0.7)	1 (0.7)	3 (1.1)	1 (0.4)	1 (0.7)	
Missing	3	0	1	1	1	
Self-reported mental health (measured using GHQ12)						0.388
Psychological distress or severe distress (GHQ12 ≥16)	127 (16.1)	22 (17.3)	44 (16.6)	45 (17.5)	16 (11.3)	
Missing	43	11	11	12	9	

% was calculated among non-missing cases. Two different cut-off points for the GHQ score were used: 20/21 (severe distress and others) and 15/16 (psychological distress or severe distress and others). A *ger* is a Mongolian traditional portable tent made from a wooden frame and covered by wool felt

*MNT* Mongolian Tugrik, *USD* US dollars

^a^ *P* value was obtained using the chi-square test

Self-reported general health differed significantly according to residential area. Subjects reporting their general health as very good or good varied according to residential area: summer house (76.3%), Mongolian *ger* (78.5%), apartment (77.6%), and luxury residence (91.9%). Subjects reporting psychological distress or severe distress, based on a GHQ12 score ≥16, varied according to residential area although statistical significant difference was not shown: summer house (17.3%), Mongolian *ger* (16.6%), apartment (17.5%), and luxury residence (11.3%).

Table 2 shows the results of a principal component analysis of responses to 23 questions regarding different aspects of the neighborhood environment. Among the 23 questions, 21 were used in the final model to exhibiting the good fitness of model. Six principle factors with an eigenvalue >1.0 explained 62.8% of the total variance in the respondents’ physical and social neighborhood perceptions. The confirmatory factor analysis showed model fitness with the following parameters: goodness of fit index (GFI) = 0.923, comparative fit index (CFI) = 0.920, and root mean square error of approximation (RMSEA) = 0.061.

**Table 2:** Principal component analysis of perceptions of neighborhood environment in Ulaanbaatar (*n* = 754)

	Principal component factor loadings^a^					
	PE factor	DE factor	NC factor	PS factor	NE factor	CS factor
Air is clean during the summer season	0.720	0.068	0.098	0.191	−0.075	0.090
Streets are clean	0.693	0.221	0.080	0.169	0.114	0.273
Easy to find green open spaces	0.669	0.202	0.112	0.022	0.291	0.010
Streets are easy to walk on	0.657	0.231	0.172	0.190	0.092	0.189
Soil is safe	0.642	0.207	0.108	0.180	0.264	−0.063
Air is clean during the winter season	0.601	0.266	0.182	0.113	0.277	−0.283
My neighborhood is friendly for children	0.230	0.840	0.149	0.147	0.085	0.062
My neighborhood is friendly for elderly and disabled people	0.285	0.810	0.130	0.100	0.061	0.126
There is enough car parking near my home	0.183	0.727	0.149	0.105	0.140	0.039
Neighbors are friendly	0.049	0.186	0.770	0.098	0.007	0.076
Neighborhoods schools provide standard education	0.016	0.117	0.683	0.088	0.056	0.383
My neighborhood is peaceful	0.297	0.019	0.611	0.068	0.223	0.071
Traffic is not bad	0.350	0.375	0.601	0.152	0.079	−0.073
Drinking water is safe	0.175	0.076	0.030	0.797	0.070	0.167
Safe, healthy food is easily accessible	0.081	0.199	−0.023	0.766	−0.011	0.161
Crime rates are low	0.202	0.091	0.325	0.674	0.158	−0.214
People follow public rules	0.354	0.027	0.317	0.588	0.195	−0.141
River water is clean	0.166	0.121	0.131	0.100	0.838	0.049
Nature is well-preserved	0.225	0.107	0.064	0.107	0.826	0.114
Family health center is very close to home	0.006	0.153	0.064	0.042	0.192	0.700
*Khoroo* office provides opportunities for participation plans/events	0.238	−0.015	0.325	0.067	−0.080	0.582
*r* ^2^ value	33.0	7.6	7.3	6.3	5.3	5.1

*Khoroo* is a community administrative unit in Mongolia

*PE*, quality of physical environment, *DE* quality of designed environment, *NC* quality of neighborhood community, *PS* quality of public safety, *NE* quality of natural environment, *CS* quality of citizen services

^a^Factors with eigenvalue >1 are shown

According to the factor loadings, these six factors were interpreted to represent (1) quality of the physical environment (PE), reflecting clean air, green spaces, cleanliness and walkability; (2) quality of the designed environment (DE), reflecting safety and accessibility; (3) quality of neighborhood community (NC), reflecting friendliness of neighbors and education; (4) quality of public safety (PS), reflecting order of public rules, less crime, and safety of food and water; (5) quality of natural environment (NE), reflecting nature preservation; and (6) quality of citizen services (CS), reflecting access to a family health center and community participation. Cronbach’s alpha of each factor was 0.721 for PE factor, 0.738 for DE factor, 0.738 for NC factor, 0.763 for PS factor, 0.780 for NE factor, and 0.799 for CS factor.

Table 3 shows the mean factor scores for perception of neighborhood environment according to residential area, education, and household income. All six factor scores differed significantly according to residential area. The perceived quality of the physical environment (PE) was high for the summer house area, while the perceived quality of the designed environment (DE) was high for the luxury residential area. The perceived quality of neighborhood community (NC) was high for summer house area, while the perceived quality of public safety (PS) was low. The perceived quality of citizen services (CS) was low for both the summer house area and the luxury residential area. Regarding both education and perceived neighborhood environment quality, there were significant differences in the DE scores according to education level. Significant differences in the PE, DE, and CS scores were observed according to the household income level.

**Table 3:** Mean factor score of perception of neighborhood environment according to residential area, education, and household income

Characteristics	PE factor	DE factor	NC factor	PS factor	NE factor	CS factor
	Mean (SD)	Mean (SD)	Mean (SD)	Mean (SD)	Mean (SD)	Mean (SD)
Residential area						
Summer house area	0.78 (0.89)	−0.08 (1.07)	0.21 (1.01)	−0.24 (0.91)	0.26 (1.08)	−0.32 (0.99)
*Ger* area	−0.34 (0.93)	−0.18 (0.86)	−0.09 (1.06)	0.14 (0.99)	−0.25 (0.95)	0.18 (1.09)
Apartment area	−0.18 (0.91)	−0.18 (0.94)	−0.02 (0.99)	−0.05 (1.00)	0.31 (0.99)	0.12 (0.89)
Luxury residential area	0.30 (0.94)	0.75 (0.94)	−0.05 (0.86)	0.02 (1.04)	0.01 (0.94)	−0.29 (0.87)
*P* value	<0.001	<0.001	0.042	0.005	<0.001	<0.001
Education						
Basic education	−0.22 (1.14)	−0.23 (0.93)	−0.05 (1.14)	−0.16 (1.11)	0.07 (1.03)	−0.05 (1.18)
High school	−0.01 (0.95)	−0.20 (0.92)	0.06 (1.06)	0.00 (0.93)	0.03 (0.93)	0.04 (0.93)
Vocational education	−0.02 (1.03)	−0.21 (0.92)	0.04 (0.88)	−0.07 (0.94)	−0.10 (0.96)	0.04 (1.02)
College or university	0.09 (0.98)	0.19 (0.99)	−0.05 (1.01)	0.10 (1.02)	−0.01 (1.04)	0.08 (0.93)
Graduate school	0.04 (0.91)	0.46 (1.13)	−0.02 (0.79)	−0.02 (1.03)	−0.01 (1.06)	−0.35 (1.07)
*P* value	0.139	<0.001	0.770	0.211	0.764	0.014
Household income, monthly						
<500,000 MNT (<250 USD)	−0.06 (1.13)	−0. 17 (0.97)	−0.11 (1.11)	0.03 (1.06)	−0.04 (1.11)	0.12 (1.11)
≥500,000 and <800,000 MNT (≥250 and <400 USD)	−0.14 (0.93)	−0.29 (0.95)	−0.01 (1.04)	−0.14 (1.01)	−0.02 (0.90)	0.11 (1.11)
≥800,000 and <1,200,000 MNT (≥400 and <600 USD)	−0.04 (0.99)	−0.03 (0.92)	0.18 (1.00)	0.05 (0.88)	−0.04 (0.95)	−0.14 (0.85)
≥1,200,000 and <2,500,000 MNT (≥600 and <1240 USD)	0.07 (0.90)	0.16 (0.98)	−0.09 (0.91)	0.11 (1.06)	0.10 (1.03)	0.05 (0.89)
≥2,500,000 MNT (≥1240 USD)	0.47 (1.02)	0.89 (0.89)	0.01 (0.78)	0.02 (0.98)	0.05 (1.06)	−0.33 (0.87)
*P* value	<0.001	<0.001	0.068	0.151	0.710	0.002

*PE* quality of physical environment, *DE* quality of designed environment, *NC* quality of neighborhood community, *PS* quality of public safety, *NE* quality of natural environment, *CS* quality of citizen services. *P* values were calculated as using ANOVA

Table 4 shows the results of a logistic regression analysis examining the association between perceived neighborhood environment and self-reported general health. In adjusted models, ORs controlling the influence of education, income, occupation, type of residential area, and number of years living in the current *khoroo* were presented. The perceived quality of citizen services (CS) in the neighborhood was associated with a better self-reported general health, independent of income, education, and type of residential area, respectively. The adjusted logistic regression analysis showed no statistical association between residential area type and self-reported health.

**Table 4:** Association between perceived neighborhood environment and self-reported general health (*n* = 719)

Neighborhood environment quality	Unadjusted	Adjusted
	OR (95% CI)	OR (95% CI)
PE factor score	1.120 (0.938, 1.337)	1.050 (0.855, 1.290)
DE factor score	1.280 (1.071, 1.531)*	1.076 (0.881, 1.316)
NC factor score	0.989 (0.837, 1.920)	1.030 (0.856, 1.238)
PS factor score	1.154 (0.968, 1.376)	1.133 (0.939, 1.368)
NE factor score	0.942 (0.789, 1.125)	0.942 (0.776, 1.142)
CS factor score	1.258 (1.050, 1507)*	1.330 (1.093, 1.618)**

*P* values were calculated using a logistic regression analysis. Odds ratios were adjusted for the education, income, occupation, type of residential area and number of years living in the current *khoroo* were calculated separately for each neighborhood environment quality.

*PE* quality of physical environment, *DE* quality of designed environment, *NC* quality of neighborhood community, *PS* quality of public safety, *NE* quality of natural environment, *CS* quality of citizen services, *OR* odds ratio, *CI* confidential interval

*P < 0.05; **P < 0.01

Table 5 shows the results of a generalized linear regression analysis and logistic regression analysis examining the association between perceived neighborhood environment and mental health as measured using the GHQ12, adjusted for education, income, occupation, type of residential areas and living year in the current *khoroo*. The results showed that the perceived quality of the neighborhood community (NC) and the perceived quality of public safety (PS) in the neighborhood were less likely to be associated with “psychological distress or severe distress,” independent of education, income, occupation, type of residential areas, and living year in the current *khoroo*. Neither the generalized linear regression analysis nor the adjusted logistic regression analysis showed any statistical association between the residential area type and mental health.

**Table 5:** Association between perceived neighborhood environment and self-reported mental health (*n* = 719)

Neighborhood environment quality	GHQ12 Likert scale score (0–36)		GHQ12 binary scale	
	Unadjusted	Adjusted	Psychological distress or severe distress (GHQ ≥16)	
	*B* (SE)	*B* (SE)	Unadjusted OR (95% CI)	Adjusted OR (95% CI)
PE factor score	−0.305 (0.187)	−0.315 (0.204)	1.124 (0.778, 1.626)	0.917 (0.738, 1.140)
DE factor score	−0.373 (0.187)	−0.214 (0.201)	0.852 (0.700, 1.037)	0.910 (0.734, 1.127 )
NC factor score	−0.598 (0.188)***	−0.572 (0.190)***	0.875 (0.720, 1.063)	0.879 (0.721, 1.073)
PS factor score	−0.733 (0.187)***	−0.720 (0.188)***	0.712 (0.586, 0.866)***	0.718 (0.589, 0.876)***
NE factor score	0.135 (0.188)	0.075 (0.190)	0.997 (0.819, 1.214)	1.004 (0.822, 1.225)
CS factor score	0.117 (0.188)	0.098 (0.194)	0.964 (0.791, 1.174)	0.941 (0.771, 1.148)

*P* values for GHQ12 Likert scale were obtained using a generalized linear regression and were adjusted for education, income, and type of residential area. *P* values for GHQ binary scale (cut-off point 15/16) were calculated using a logistic regression analysis. Odds ratios were adjusted for education, income, occupation, type of residential area, and number of years living in the current *khoroo* were calculated separately for each neighborhood environment quality

*PE* quality of physical environment, *DE* quality of designed environment, *NC* quality of neighborhood community, *PS* quality of public safety, *NE* quality of natural environment, *CS* quality of citizen services, *B* standardized coefficient, *SE* standard error, *OR* odds ratio, *CI* confidential interval

***P < 0.001

## Discussion

This study examined how middle-aged women living in rapidly urbanizing Ulaanbaatar perceived their neighborhoods and how these perceptions related to their self-reported general health and mental health. Variations in self-reported general and mental health were not significantly associated with living in a particular residential area type in Ulaanbaatar after excluding influence of socio-economic status of women. The self-reported general health status was associated with the quality of citizen services, and the self-reported mental health status was associated with the quality of public safety. The results revealed several complexities in how people think about what constitutes a healthy neighborhood.

Among the six components of neighborhood quality identified in this study, four of them were similar to qualities that Araya et al. previously identified in a study conducted in the UK [13]. These components were “designed environment,” “neighborhood community,” “public safety,” and “citizen services.” Another study conducted in Tokyo [12] identified perceptions of the quality of the physical environment. The “physical environment” component identified in the present study is similar to this previously reported concept. How people recognize their neighborhood environment is influenced by social norms, lifestyle, and the physical quality of environment. Therefore, for the time being, patterns of perceived neighborhood quality by residents should be carefully identified based on the responses of people living in particular individual settings.

In this study, “citizens’ services,” a factor related to women’s general health, refers to the respondents’ perceived access to a family health center, as well as the extent of opportunities for community participation in plans and events within the neighborhood. These findings are consistent with two studies from Brazil demonstrating links between the quality of public services and better self-reported health [5, 16]. Prioritizing the early availability of good citizens’ services in new neighborhoods may help deliver better self-reported general health for their residents.

Previous international studies have identified a wide range of neighborhood environmental factors that are associated with self-reported general health: neighborhood problems [17], sociability [18], neighborhood amenities [19], the physical quality of residential areas [20], fewer physical and social disorders [5], the neighborhood’s physical environment [4], social cohesion and a general feeling of safety [21], and social safety [22]. While our results, which focus on middle-aged women in urban neighborhoods, did not identify some of these relationships, each of these factors has a potential impact on self-reported health in different contexts and populations in Mongolia and should be considered in future studies.

The mental health status, as measured using the GHQ12, was found to be primarily associated with two specific neighborhood qualities: neighborhood community and public safety. Neighborhood community incorporates the friendliness of neighbors, the local education availability for children, and a feeling of peacefulness in the neighborhood (including in terms of traffic). In this analysis, “public safety” was defined as follows: a low perceived crime rate, general public obedience to rules, plus people’s access to safe food and water in the neighborhood. Previous studies that have specifically considered mental health in the context of the neighborhood environment have mostly been conducted in developed countries. They demonstrated significant associations between the GHQ score and social cohesion, trust [13], and social relationships [23]. This study, which focuses on the capital city of a low–middle-income country, indicated not only social relationship in the community but also residents’ perception on public safety was associated with their mental health.

The perceived friendliness of neighbors is of particular resonance in Mongolian culture which has a society that is currently transitioning from a traditionally nomadic way of life to an urban setting. In the former setting, families knew each other intimately, while in the current setting, families may no longer know even their closest neighbors. Relatedly, the crime rate is rising in Ulaanbaatar, with theft accounting for 32.9% of all crime in the city [24]. This trend has led people to become increasingly anxious and distrustful of their neighbors. Promotion of public safety in transitioning society is crucial for good mental health of the population.

Surprising, this study did not find a specific correlation between access to green spaces and mental health status, despite substantial international evidence to support this link [25–27]. However, in this aspect, our analysis may not reveal the full picture. A study from Sweden similarly showed no associations between wild, lush, spacious natural environments and mental health, but rather found associations with elements of peace and silence [28]. Further study examining the importance of green spaces is needed in the context of Mongolian topography and lifestyles.

Since the data was obtained using an interview survey with a cross-sectional design, we were unable to exclude a potential reporting bias or to address causalities. Particularly, how current perceptions of the neighborhood environment will be correlated with interactions with the environment and the health of individuals during their later life should be a topic of investigation in future studies. Urbanization in Ulaanbaatar has topographic and socio-political characteristics that are specific to Mongolia. Its location in north-central Asia, the socio-political changes that it has undergone in transitioning from a socialist to a free-market economy, and its cold climate and limited vegetation create a specific background for life in Ulaanbaatar. However, knowledge of the changes in residential environments and their relationship with general and mental health in the context of urbanization is of relevance to other countries, particularly those with similar socio-economic and political settings.

With the recent rapid economic and population growth that Ulaanbaatar has experienced, neighborhoods with modern housing and high-quality amenities are now emerging. People living in these new areas tend to be well educated and to have higher household income levels and good self-reported health. However, with these developments come numerous environmental problems associated with urbanization, such as indoor and outdoor air pollution [29, 30], waste management, water quality, indoor energy use [31], and automobile traffic [32]. As the city develops, projections of the scale of these problems are needed to enable planning to minimize the negative impacts of urbanization on the health of its residents.

Alongside development and redevelopment efforts to improve physical neighborhood environments, this study highlights the importance of developing the social environment and opportunities for local residents. In Ulaanbaatar, better self-reported general health was associated with good access to supportive and engaged community services with good education and economic opportunities. Better self-reported mental health was associated with safe, friendly, supportive, engaged, and inclusive communities with good education and economic opportunities. These pro-health neighborhood environments occur across residential area types and income levels in Ulaanbaatar. The shared qualities offer important lessons for urban planning and design—particularly in the context of rapidly developing cities. The creation of successful, healthy neighborhoods requires more than physical infrastructure: strategic planning to deliver good social services, community, and safety in urban neighborhoods will support better health and well-being and help to create the cities that we need.

## Conclusions

The perception of the quality of a neighborhood environment can affect the self-reported general and mental health of residents, even after accounting for the type of residential area and individual socio-economic status. Results of a survey conducted in rapidly urbanizing Ulaanbaatar, Mongolia, showed that the self-reported general health status of middle-aged women was associated with the quality of citizen services and their self-reported mental health status was associated with the quality of public safety. The results revealed several complexities in how people think about what constitutes a healthy neighborhood. Developing high-quality neighborhoods is an essential component of good planning to promote population health in urban environments.

## Abbreviations

CFI: Comparative fit index
CI: Confidence interval
CS: Citizen services
DE: Designed environment
GDP: Gross domestic product
GFI: Goodness of fit index
GHQ12: 12-item General Health Questionnaire
NC: Neighborhood community
NE: Natural environment
OR: Odds ratio
PE: Physical environment
PS: Public safety
RMSEA: Root mean square error of approximation
USD: US dollars
